# Crystal Structure of the PAC1R Extracellular Domain Unifies a Consensus Fold for Hormone Recognition by Class B G-Protein Coupled Receptors

**DOI:** 10.1371/journal.pone.0019682

**Published:** 2011-05-19

**Authors:** Shiva Kumar, Augen Pioszak, Chenghai Zhang, Kunchithapadam Swaminathan, H. Eric Xu

**Affiliations:** 1 Laboratory of Structural Sciences, Van Andel Research Institute, Grand Rapids, Michigan, United States of America; 2 Department of Biological Sciences, National University of Singapore, Singapore; University of Oldenburg, Germany

## Abstract

Pituitary adenylate cyclase activating polypeptide (PACAP) is a member of the PACAP/glucagon family of peptide hormones, which controls many physiological functions in the immune, nervous, endocrine, and muscular systems. It activates adenylate cyclase by binding to its receptor, PAC1R, a member of class B G-protein coupled receptors (GPCR). Crystal structures of a number of Class B GPCR extracellular domains (ECD) bound to their respective peptide hormones have revealed a consensus mechanism of hormone binding. However, the mechanism of how PACAP binds to its receptor remains controversial as an NMR structure of the PAC1R ECD/PACAP complex reveals a different topology of the ECD and a distinct mode of ligand recognition. Here we report a 1.9 Å crystal structure of the PAC1R ECD, which adopts the same fold as commonly observed for other members of Class B GPCR. Binding studies and cell-based assays with alanine-scanned peptides and mutated receptor support a model that PAC1R uses the same conserved fold of Class B GPCR ECD for PACAP binding, thus unifying the consensus mechanism of hormone binding for this family of receptors.

## Introduction

G-Protein Coupled Receptors (GPCRs) are important regulators of many physiological functions and as such have attracted a lot of pharmacological interest for their roles in numerous diseases. Pituitary adenylate cyclase 1 Receptor (PAC1R), which belongs to class B of the GPCR family, was identified in 1994 as the receptor for the peptide hormone Pituitary Adenylate Cyclase Activating Polypeptide (PACAP) [1]. PACAP was discovered earlier as an adenylate cyclase stimulating agent in ovine hypothalamus [2]. Due to the fact that PACAP is homologous to another peptide hormone, Vasoactive Intestinal Peptide (VIP), there is redundancy in their receptors, which are sub-classified based on the differences in their relative affinities for the two hormones. The receptors that exhibit similar affinity for both PACAP and VIP are classified as VPACR [3], [4] while those showing higher affinity selective for PACAP over VIP [3], [4], [5] have been classified as PAC1R. VPACR has further been subdivided based on its affinity for helodermin [6], which is a bioactive peptide that was first isolated from the poisonous salivary gland secretions of the gila monster (*Heloderma suspectum*) [7]. Helodermin, a member of the exendin family of peptides and sequentially related to the PACAP/glucagon family, is present exclusively in the gila monster [8].

Since its discovery as an activator of adenylate cyclase in cultured sheep pituitary cells [2], PACAP has been found in numerous locations in the central nervous system as well as peripheral organs [8], [9]. PACAP and its receptor PAC1R have been implicated to play important roles in several cellular processes, including regulation of circadian rhythm, control of food intake, glucose metabolism, learning and memory, neuronal ontogenesis, apoptosis, and immune system regulation. Furthermore, PACAP and the PAC1 receptor have recently been implicated in post-traumatic stress disorder [10]. As such, PAC1R has been pursued as a drug target for numerous disorders, including neuropathic analgesia [11], septic shock [12], islet dysfunction in type 2 diabetes [13], and Parkinson's disease [14]. PACAP has been conserved over 700 million years [8], which indicates its critical functions in vertebrate physiology. Indeed, 60% of PAC1R knockout mice die within 4 weeks after birth [15]. The varied functions of PAC1R become even more intricate by the opposing roles of PACAP in different tissue types [16], [17] [18]. While PACAP has been shown to have neurotrophic properties [16], [17], it has also been suggested to be involved in apoptosis and cell cycle arrest in different systems [18]. PACAP exists in two forms: PACAP38 and its shorter version PACAP27. The relative abundance of these two forms in tissues and their subtle differences in binding to the receptor [19], [20] also contribute to the opposing roles of the receptor. This makes PAC1R a very intriguing relay junction and structural studies of how PACAP binds to PAC1R will provide a basis for understanding signaling pathways by these peptide hormones.

As a member of Class B GPCR, PAC1R uses a “two-domain” mechanism for hormone binding and activation [21]. Its ECD is the major hormone binding site that determines specificity and affinity through the binding to the C-terminal portion of peptide hormones. Upon binding to the ECD, the N-terminal portion of peptide hormone interacts with the transmembrane domain and activates the receptor. In addition, a growing number of evidences suggest that receptor activity modifying proteins (RAMPs) may be involved in modulating signalling at the extracellular surface for the subfamily of calcitonin receptors [22], [23]. One of the major characteristics of the Class B GPCR ECD is the three-conserved disulfide bonds that form the core of the ECD structure. Crystal structures of ECD and hormone/ECD complexes have been recently determined for several Class B GPCRs, including receptors for parathyroid hormone (PTH) [24], glucose-dependent insulinotropic peptide (GIP) [25], glucagon-like peptide 1 (GLP1) [26], corticotropin-releasing factor (CRF) [27], [28], [29], and calcitonin gene-related peptide receptor [23]. These structures reveal a common scaffold for the Class B GPCR ECD and a helical structure for the bound peptide hormone, suggesting a consensus model of hormone binding in Class B GPCRs. However, the mechanism of how PACAP binds and activates its receptor remains speculative and controversial as an NMR structure of the PAC1R ECD/PACAP complex reveals a different topology of the ECD and the mode of ligand recognition [30]. To resolve this discrepancy, we performed biochemical and structural studies with the PAC1R ECD. The crystal structure of the PAC1R ECD unifies a conserved and consensus fold among all known Class B GPCR structures.

## Materials and Methods

### Protein production

The extra-cellular domain of the human PAC1R protein (residues 25–140 of isoform 4, NCBI reference sequence: NP_001186566.1, having an exon deletion in N-terminal ECD) was overexpressed as a maltose binding protein (MBP) fusion protein. The MBP tag was at the N-terminus, separated from PAC1R by a six residue linker (NAAAEF) and a 6xHis tag was attached at the C-terminus to ensure affinity purification of only the full length protein. The MBP-PAC1R construct was cloned into the pET-Duet1 vector having the disulphide bond chaperone (DsbC) at the second multiple cloning site, in order to facilitate proper disulphide bond formation during protein over-expression in bacteria. Origami B (DE3) cells (Novagen), transformed with the construct, were grown in 6 L selective LB media (50 µg/ml carbenicillin) at 37°C until mid log phase, then cooled to 16°C and induced with 200 µM IPTG for 18 hrs. The cells were harvested by centrifugation at 3000 g, re-suspended in buffer A [50 mM Tris (pH 7.5), 150 mM NaCl, 25 mM imidazole and 10% glycerol] and lysed in a French press at 10,000 psi. The cell lysate was centrifuged at 39,000 g for 20 minutes and the supernatant was loaded onto a Ni chelating Sepharose column (35 ml resin in a 50 ml column). The column was then washed with 600 ml buffer A and then the bound protein was eluted with 50% buffer A and 50% buffer B [50 mM Tris (pH 7.5), 150 mM NaCl, 500 mM imidazole and 10% glycerol]. The eluted sample was then loaded onto an amylose column (30 ml resin in a 50 ml column). The column was then washed with 150 ml buffer C [50 mM Tris (pH 7.5), 150 mM NaCl] and then subsequently the protein was eluted using a gradient of buffer C and buffer D [50 mM Tris (pH 7.5), 150 mM NaCl, 10 mM maltose] from 0 to 100% over 200 ml volume.

To ensure homogenous intramolecular disulphide bond formation, the eluted protein was subjected to disulphide bond shuffling, in which, the eluted protein was mixed with purified DsbC in 1∶1 molar ratio in addition to 1 mM reduced and 1 mM oxidized glutathione at 20°C for 12–14 hrs. The shuffled protein was then loaded onto the 50 ml Ni chelate column to remove untagged DsbC. After washing the column with 150 ml buffer A, the bound protein was eluted with 50% buffer B. The protein was then concentrated and loaded onto an S200 column, equilibrated with buffer E [10 mM Tris (pH 7.5), 50 mM NaCl, 1 mM maltose and 1 mM EDTA] for size exclusion chromatography. The obtained protein was >95% pure and homogenously folded as confirmed by SDS- and native PAGE.

PACAP38-MBP was expressed in modified pSUMO vector (TOP Gene Technologies) in which the MBP gene had been introduced c-terminal to Bsa I site. The H6-SUMO-PACAP-MBP polypeptide was expressed in BL21(DE3) cells. The cell pellet was lysed in buffer A and the supernatant incubated with Ni-chelating sepharose beads. The beads were then washed with buffer A and the protein eluted in buffer B. To make the N-terminal of PACAP available for interaction with the juxtamembrane region of full length receptor the SUMO tag was cleaved by adding SUMO protease at 1∶1000 mass ratio. After overnight incubation with the protease the buffer was exchanged back to buffer A using 0.5 ml Amican Ultra 10 Kda centrifugal filters. The lysis mixture was incubated again with Ni-chelating sepharose beads to remove the SUMO tag. The final yield of PACAP38MBP wild-type and all mutant proteins was ∼4.5 mg/L of culture.

### Crystallization, data collection and structure determination

The purified PAC1R protein was concentrated to 30 mg/ml and screened for crystallization using commercial screens (Hampton Research) by a Phoenix robot (Art Robbins Instruments). The final crystals for data collection were grown in hanging drop plates at 20°C, 25.5% PEG4000, 15% glycerol and 170 mM ammonium sulphate. Crystals first appeared after three days and grew for one week, after which they were transferred to a fresh drop and allowed to equilibrate by vapour diffusion against 1 ml of reservoir solution overnight. The crystals were then flash cooled, without any additional cryo-protectant, by plunging directly in liquid nitrogen. X-ray diffraction data were collected at the LS-CAT sector beamline 21ID-F (LS-CAT), Advanced Photon Source synchrotron (USA). The data were processed by HKL2000 [31] and the structure was solved by molecular replacement using the previously reported MBP-PTH1R structure (PDBId: 3C4M) as a model in the PHASER program [32] of CCP4 [33], [34]. Coot [35] and Refmac5 [36] were subsequently used for iterative model building and refinement, respectively. The geometry of the final model was verified using PROCHECK [37]. No residues in the final model are in disallowed region of Ramachandran plot. The crystallographic details are given in Table 1.

**Table 1:** Data collection, structure determination and refinement details.

Space group	P2_1_2_1_2_1_
Unit-cell parameters (Å):	a = 45.98
	b = 92.15
	c = 105.41
**Data collection**	
Wavelength (Å)	0.98972
Resolution range (Å)	50.0-1.9
Total no. of observed reflections	579,761
Total no. of unique reflections	42,645
Redundancy	13.6 (12.0)
Completeness (%)	100 (100)
R_sym_ a	0.090 (0.570)
Overall I/σ(I)	28.8 (4.7)
**Structure determination**	
Method	Molecular replacement
Model	3C4M
**Refinement**	
Resolution range (Å)	50.0-1.9
R_work_ b	0.176
R_free_ c	0.216
RMSD bond lengths (Å)	0.020
RMSD bond angles (°)	1.7
Average B-factors (Å^2^):	
Protein atoms (5724 atoms)	34.04
Water molecules (323 atoms)	35.35
Ramachandran plot:	
Most favored regions (%)	93.2
Additional allowed regions (%)	6.8

Values in parentheses are for the last shell (1.98-1.9 Å).

a R_sym_ = Σ_hkl_ Σ_i_ [|I_i_ (hkl)−<I(hkl)>|]/Σ_hkl_ I_i_(hkl).

b R_work_ = Σ|F_obs_−F_calc_|/Σ|F_obs_| where F_calc_ and F_obs_ are the calculated and observed structure factor amplitudes, respectively.

c R_free_ is same as R_work_, but 5.0% of the total reflections, chosen at random, were omitted during refinement.

### Peptide binding assay

The binding of PAC1R with a 35 residue PACAP peptide was analysed using the histidine detection kit of AlphaScreen® luminescent proximity assay from PerkinElmer. All binding assays were performed without cleaving the MBP tag from the receptor ECD. Using its C-terminal His tag, PAC1R was bound to Ni-chelate coated acceptor beads (5 µg/ml) at the final concentrations of 40 nM. Similarly, biotinylated PACAP was bound to streptavidin coated donor beads (5 µg/ml) at the final concentrations of 40 nM. The protein and peptide were pre-incubated with the respective beads for one hour (to ensure maximal binding to the beads), then they were allowed to interact with each other at 20°C for five hours. To perform competitive binding assays, 120 µM of the untagged competing peptides were added at time 0 of the five hour protein-peptide interaction period. All interactions were performed in the background of 50 mM MOPS pH 7.4, 150 mM NaCl and 7 mg/ml BSA. The binding signals were measured in a 384-well microplate using an Envision 2104 plate reader (PerkinElmer). The obtained data for competitive peptide binding were fit as a non-linear regression curve using the variable slope dose-response inhibition analysis using the Prism5.0 (GraphPad). To ensure the specificity of the competing peptides, a control peptide of biotin-Gly6-His6 was used in the same assay setup. The competing peptide, at concentrations even higher than those used in the actual assay, does not affect the binding signal of the control peptide.

### Docking of PACAP8–27 to PAC1R-ECD

The coordinates of PACAP (residues 8–27, hereafter PACAP8–27) were obtained from the NMR structure of the micelle bound form of the peptide (PDB code:2d2p). A course grid covering the entire PAC1R-ECD was used to do a blind search for the binding site of the rigid peptide. The grid box was kept large enough to allow Autodock 4.2 [38] to search all possible ECD interacting orientations. Lamarkian genetic algorithm (GA) was used as the search algorithm to locate the peptide docking site [39]. 100 GA runs were performed before clustering the obtained solutions. The top cluster having rank 1 comprised of 23/100 solutions, the highest among the 10 obtained clusters. In this cluster, PACAP was positioned at the same site as in other Class B GPCR:ligand complex structures. The grid was then made finer and smaller. The new grid was still made to cover the entire face of ECD where PACAP docked in the first run. The bonds in the side chains of K20′ and Y13′ in PACAP8–27 were made rotatable to allow for a better fit. This yielded a total of 7 rotatable bonds. The new run was performed using a search criterion similar to the first run except that the number of GA runs was raised to 200. Guided by the knowledge of the other ECD:peptide complex structures, a cluster with rank 5 (among 17) was chosen as the proposed model.

### cAMP signalling assay

PAC1R stimulation by PACAP38MBP was assayed using the Dual Luciferase Reporter Assay System from Promega. AD293 cells were transfected with full length receptor in pcDNA3.1 (25 ng), CRE-luciferase in pGL4.29 (100 ng) and phRL-TK (5 ng) plasmids. Receptor was stimulated for 4 hours by adding PACAP38-MBP at 37°C. The cells were then lysed and CRE luminescence quantified in 96-well plate using Envision 2104 plate reader (PerkinElmer) using the manufacturer's instructions. *Renilla* luciferase, expressed using phRL-TK vector, was used as internal control.

## Results

### Disulphide shuffling

**Figure 1:**
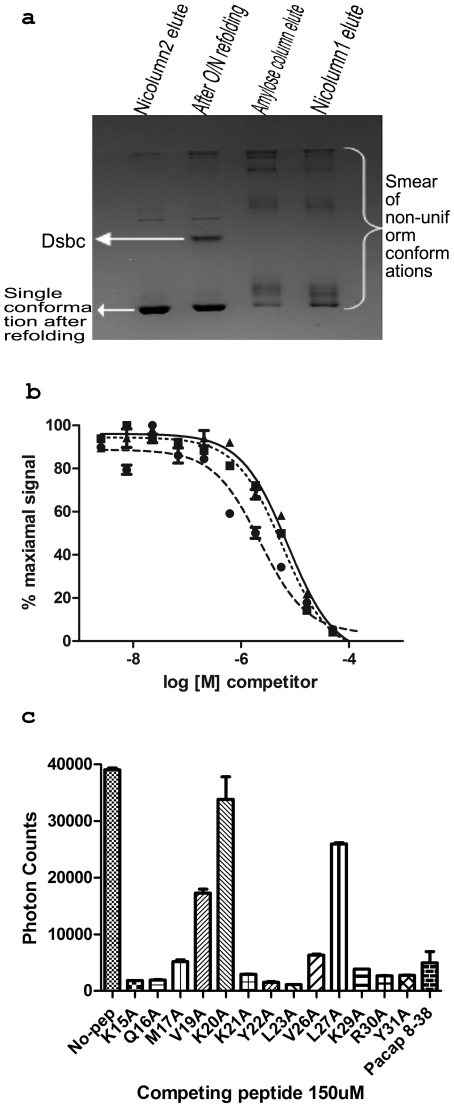
Biophysical characterization of PAC1R. (**a**) Non-reducing native PAGE of PAC1R. MBP-PAC1R gives a smear at the initial steps but attains a homogenous conformation after refolding. DsbC, which appears as an extra band after refolding, was removed by Ni Column-2 purification. (**b**) Competitive Alphascreen with unlabelled PACAP as competitor for binding of 40 nM biotin-PACAP (6–38)-NH2 and 40 nM MBP-PAC1R (25–140)-His6 in the presence of 5 µg/ml beads. Dashed, dotted and solid curves represent un-labeled PACAP (8–38), PACAP (12–27) and PACAP (15–31) as competitors, respectively. (**c**) Binding affinities of different Ala scanning mutants of PACAP (15–31). K20A′ mutation completely abrogates PACAP binding while L27A′ and V19A′ also seem to play important roles in the binding. PACAP residues 18, 24, 25 are Ala and 28 is Gly and, therefore, were not mutated to another residue.

The ECD of human PAC1R (25–140), which sequence is shown in Fig. S1, was expressed in the Origami B (DE3) cell. In order to allow the protein to fold properly and enhance conformational homogeneity, we shuffled its disulphide bonds using redox conditions in the presence of DsbC, a bacterial disulfide bond isomerase, as described previously [24]. The gel filtration profile gave a comparatively much larger peak at the size of a monomer and relatively small (approximately 20% of the larger peak) peak at a very high molecular weight range, which, when analysed on native PAGE, appeared to be heterogeneous high molecular weight aggregates. The conformational homogeneity of the final protein preparation and its relative improvement during the steps of purification and disulphide shuffling was analysed in non-reducing native PAGE (Fig. 1a). It is apparent that the protein from the initial steps has multiple conformations and appears as a smear in native PAGE but after refolding it gives a single band with no smear in the native gel, suggesting the conformational homogeneity of the purified protein.

### PAC1R∶PACAP interaction

The binding activity of the purified PAC1R ECD to PACAP was determined by AlphaScreen assay, in which N-terminally biotinylated and C-terminally amidated PACAP 6–38 (hereafter called B-Pacap 6–38-NH2) was made to bind with streptavidin coated donor beads. MBP-PAC1R (25–140) with a C-terminal 6xHis tag was attached to Ni-chelate coated acceptor beads. Association of PACAP with PAC1R was analysed by Alphascreen assays, which is based on proximity transfer of excitation energy from the donor to the acceptor beads in a dose dependent manner. The Alphascreen signal was disrupted with unlabelled PACAP fragments, which still retain the essential binding region, to obtain IC_50_ values (Fig. 1b). The IC_50_ values for the truncations, including PACAP12–27 and PACAP15–31, did not change much among each other and remained in the 1–10 µM range, which is consistent with the previously reported values for other class B GPCR ECDs and their native peptide ligands [24].

### Crystal structure of PAC1R ECD

**Figure 2:**
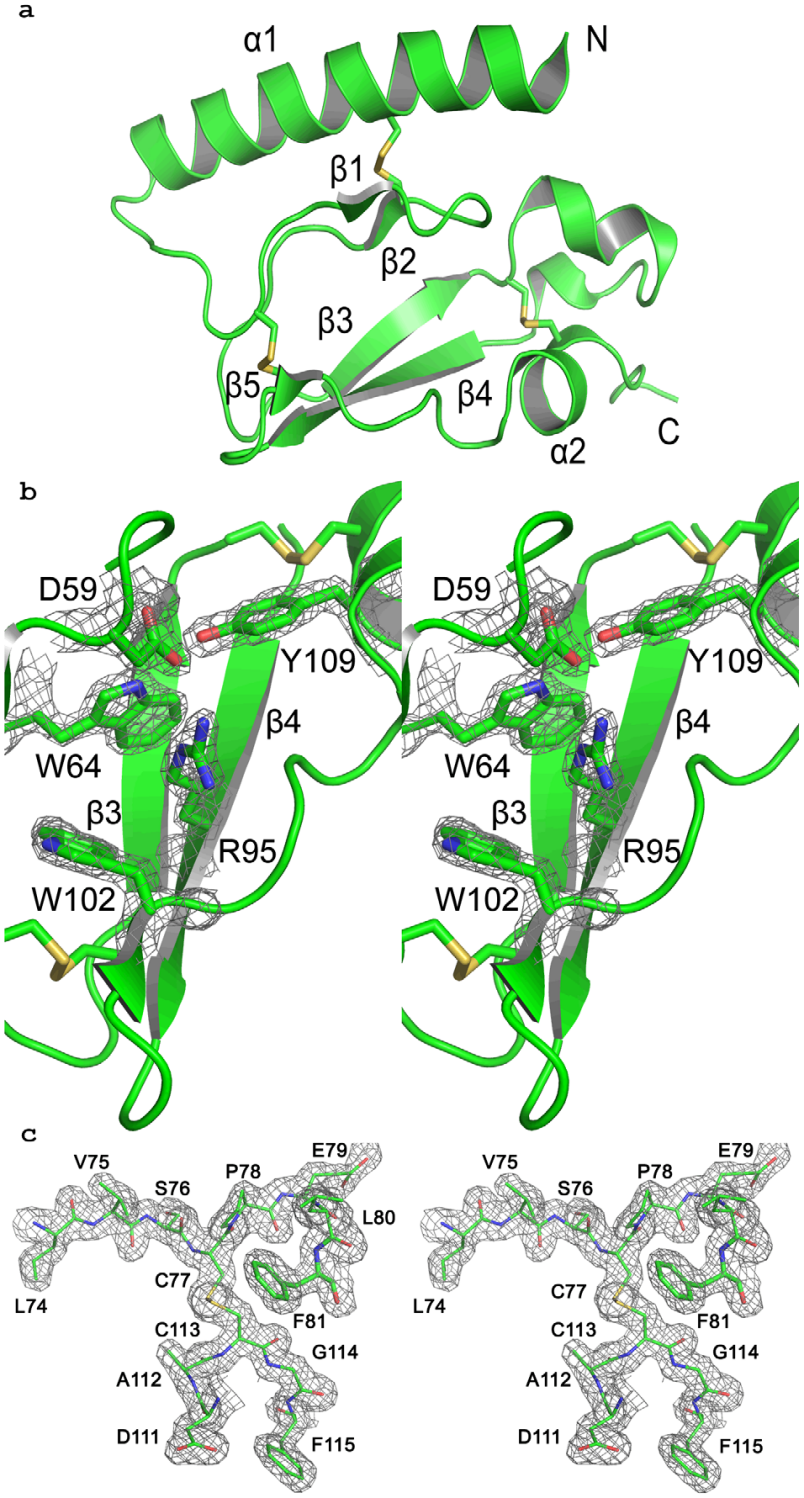
Structure of PAC1R. (**a**)(**i**) The ribbon diagram of the PAC1R ECD. Strands β1 and β2 make an anti-parallel β-sheet and strands β3, β4 and β5 make another anti-parallel β-sheet. (**b**) The stereo view of the electron density around the conserved residues that form the core of the PAC1R structure. The density is contoured at the 2σ level. (c) Stereo view of the electron density for the C77–C113 disulphide bond and the residues around it contoured at 1 σ. The residues are as labelled. The electron density obtained for the β3–β4 loop is clean allowing even the side chains to be discerned clearly.

**Figure 3:**
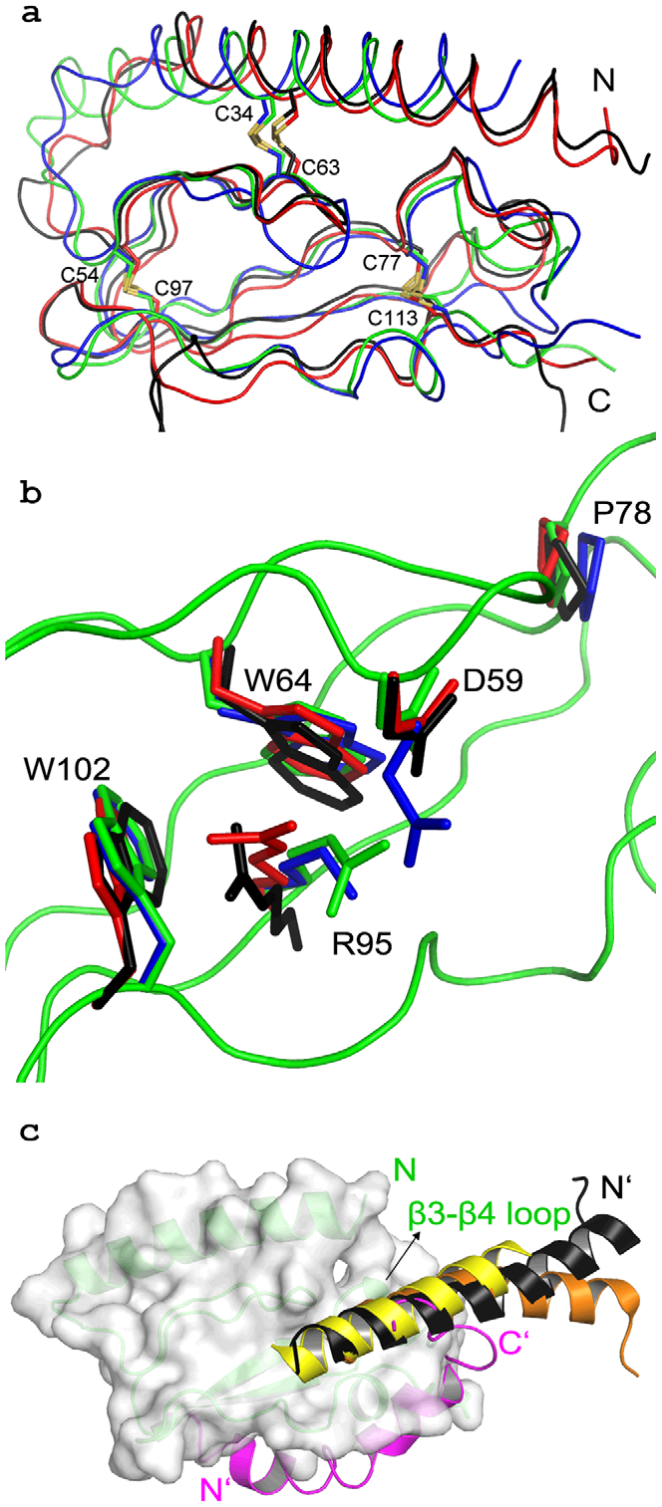
Superimposition of the backbone of the ECD of class B members. (**a**) Superimposition of the backbone of the ECD of class B members GIP1R (black, PDB code:2QKH), GLP1R (red, PDB code:3C5T), PAC1R (green, PDB code:3N94) and VIP2R (blue, PDB code:2X57). The disulphide bridges C34–C63, C54–C97 and C77–C113 are labelled. (**b**) superimposition of conserved residues. The color scheme is as in panel A. (**c**) Ligand orientation in class B GPCRs. The ligands incretin of GIP1R, glucagon of GLP1R and PTH of PTH1R are shown in black, orangeand yellow, respectively. For simplicity the surface diagram of PAC1R ECD alone is shown in white with the trace of PAC1R in green. PACAP from the NMR structure is shown in magenta. While all the class B ligands follow the same binding site and orientation, PACAP of the NMR structure is shown to bind PAC1R at a different location. Furthermore, its polarity does not follow that of the other ligands.

The crystal structure of PAC1R ECD was solved at 1.9 Å resolution with R and R_free_ of 17.6 and 20.6%, respectively. The overall crystal structure of PAC1R (Fig. 2a, S2) is very similar to other previously elucidated ECD structures of CRFR1 [27], CRFR2 [28], [29], GLP1R [26], GIP1R [25] and PTH1R [40]. The three conserved disulphide bonds hold the core of the PAC1R polypeptide together in a sandwich like configuration [25]. The N terminus starts with an α-helix that begins from Ala25 and continues until Asn48. This is followed by a short anti-parallel β sheet, held below the helix by a disulphide bridge. Then there is another anti-parallel β sheet, which is held by another disulphide bond that bridges β5 to the loop just preceding β1. The orientation of the loop between β3 and β4 relative to α-helix 2 is constrained by the third disulphide bridge between Cys77 and Cys113. The sequentially invariant amino acids (Fig. 3b), among the class B GPCR ECDs (apart from the disulphide bonded cysteins), Asp59, Trp64, Pro78, Gly101 and Trp102, are placed in the structural core of PAC1R. The structure is further stabilized by an interaction cluster formed by Asp59, Trp64, Val93, Arg95, Trp102 and Tyr109 (Fig. 2b). Most of the residues in this cluster, Asp59, Trp64, Arg95, Trp102, are highly conserved. Residues Val93 and Tyr109 of this cluster are sequentially less conserved but still appear to make important interactions. In particular, the side chain of Tyr109 points inward to contribute to hydrophobic interactions at the core through its aromatic ring while making a hydrogen bond with Asp59 through its hydroxyl group. Asp59, with its side chain directed towards the β turn below helix 1, makes crucial contacts with the backbone amides of Asn60, Ile61 and Thr62 to form a β-turn. Although the side chain of Asp59 bends towards the β-turn, it is still able to make a salt bridge with Arg95. This salt bridge is not observed in GIP1R and PTH1R. This is possible because the long side-chain of Arg95 is pushed down and towards Asp59 by the protruding side-chain of Met57. Furthermore, an interesting feature is that the bond between Phe106 and Pro107 is in the *cis* conformation. It interacts with the backbone amide of Ser94, causing a turn at Pro107 just before the beginning of helix 2, thus, constraining its orientation further, in addition to the third disulphide bridge.

Superimposition of the PAC1R crystal structure with that of other class B GPCRs (Fig. 3a) depicts that the sandwich fold is well conserved in this family, even though sequence alignment of the family members shows relatively less conservation (Fig. S1). In addition, the B-factor plot of PAC1R does not show any region of large conformational flexibility. The conserved residues overlap remarkably well in the structural alignment (Fig. 3b), depicting their importance in holding the core domain together.

### Comparison of PAC1R crystal structure with its NMR structure and the VIP2R crystal structure

**Figure 4:**
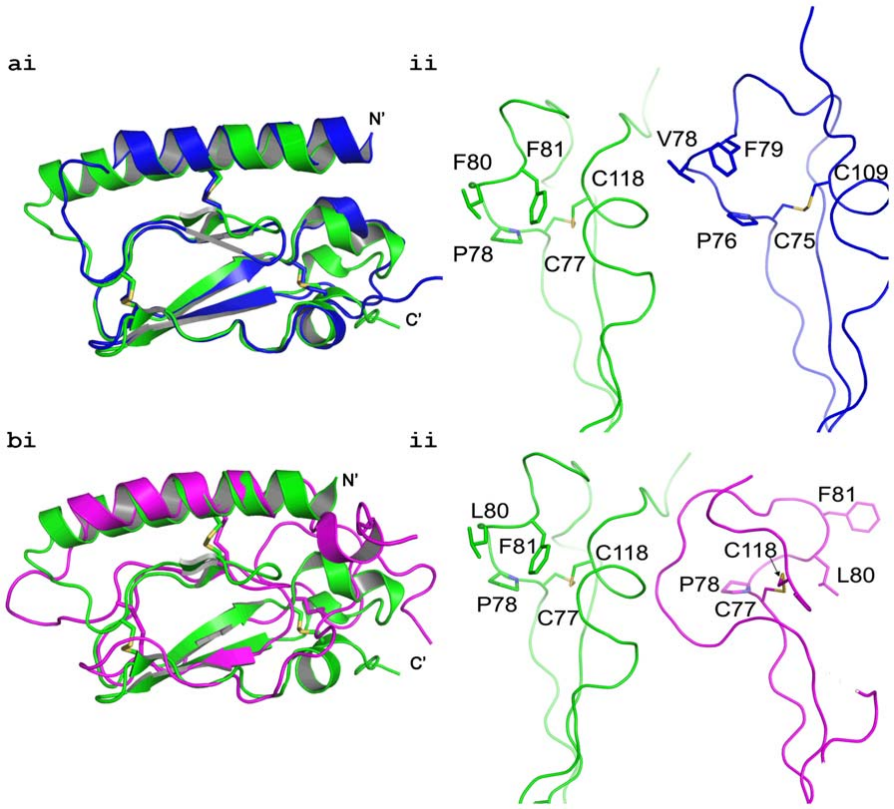
Superimposition of PAC1R/VIP2R and PAC1R X-ray and NMR structures. (**a**) the C-terminal portion of PAC1R (green) and VIP2R (blue) depicts the expected similarity in (**i**) the backbone and (**ii**) the position of the conserved residues. (**b**)(**i**) Superimposition of the backbone of the X-ray (green) and NMR (magenta) structures of the PAC1R ECD. The two molecules were aligned using Pymol and laterally separated (**ii**) the close up of selected residues in the two structures. Note the unexpected dissimilarity in the position of the conserved residues and near the C77–C118 disulphide linkage, which would warrant its disruption to allow such massive displacement in solution. Furthermore, the region around Pro78 is very different between the X-ray and NMR structures. However, this region of the X-ray PAC1R structure and the VIP2R structure superimposes well and shows the expected conformational similarity.

The VIP2R ECD structure was recently deposited to the PDB database (pdb code: 2X57). The Needleman-Wunsch protocol [41]aligns the VIP2R and PAC1R sequences with 33.1% identity and 28.8% gaps, suggesting a significant structural similarity between them. Fig. 4a clearly shows that the backbones of PAC1R and VIP2R are very similar. Their overlay is remarkably similar except at the C-terminus, where they tend to diverge slightly. However, this is expected due to high B-factors, as a result of terminal disorder. The conserved residues, as shown in Fig. 4a, are also well aligned. There is a slight deviation near Leu80 and Phe81 of PAC1R which are less conserved in the family. The two crystal structures align with an RMSD of 0.99 Å (for 64 Cα atoms), as determined by the ‘align’ routine of Pymol (The PyMOL Molecular Graphics System, Version 1.2, Schrodinger, LLC).

Similar conformational properties are also expected from the NMR PAC1R structure [30]. Surprisingly, that does not appear to be the case. In a structural alignment of class B GPCRs, the NMR-PAC1R structure stands out due to differences in the topology of the region between β3 and β4 and the arrangement of disulfide bonds. Most notably, in the NMR structure, Pro78, which is an invariant residue among the family members, is positioned very differently from the crystal structures as seen in Fig. 4b. Pro78, and its corresponding residues in the family, play important role in the structural stability of the ECD. In PAC1R, Pro78 fills the hydrophobic cavity formed by Glu30, Ile61, Thr62, Leu80 and Phe81. In the structure of Glip1R the proline residue corresponding to Pro78 has been shown to play a structurally important role in forming a ligand binding site [26]. In addition, mutation in the corresponding proline in human parathyroid hormone receptor 1 has been shown to cause embryonic lethal disorder [42]. Therefore, such variation in the conformation of Pro78, as seen in the NMR-PAC1R structure, is highly unlikely. Moreover, the alternate arrangement of the polypeptide in that region in the NMR structure, as evidenced in Fig. 4b, seems impossible because it would require the disruption of the Cys77-Cys113 disulphide bond.

The RMSD of structural alignment between NMR-PAC1R and X-ray PAC1R is 3.05 Å for (73 Cα atoms). The sequence alignment between PAC1R and VIP2R is only 33% identity and yet the RMSD between their structures is only 0.99 Å. The RMSD between the NMR and X-ray structures of PAC1R is far larger than the RMSD between the crystal structure of PAC1R and VIP2R, even though the NMR and X-ray structure of PAC1R have identical amino acids. To further evaluate these structural differences we superimposed the structures of GIP1R, GLP1R, PTH1R and VIP2R ECDs using the Superpose 1.0 web server [43] to derive an average model. Alignment of this average model with the NMR-PAC1R and X-ray-PAC1R structures gave RMSDs of 3.01 and 2.17 Å, respectively. This fact as well as Fig. 3a highlight that the fold in all class B ECDs is likely to be well conserved. Furthermore, the X-ray structure follows the consensus class B fold nicely, suggesting a unified consensus mechanism for hormone binding. The deviation of the NMR-PAC1R structure from all the published class B ECD crystal structures, which align well among themselves, implies that the accuracy of the NMR structure of the PAC1R∶PACAP complex remains to be validated.

### Important residues for PAC1R-PACAP interaction

Despite numerous attempts, even with various lengths of PACAP, we could not obtain a complex crystal. Hence we built a model of the PAC1R ECD/PACAP(12–27) based on the structure of the GLP1R/GLP1 complex as PACAP and GLP1 share a great degree of similarity. PAC1R-ECD has a predominance of negatively charged residues. In fact, it is interesting to note that the sequence has significant abundance of both Asp and Glu. The opposite is true for its ligand where the most abundant amino acid is Lys while Arg is the second most abundant. This suggests that their interaction might be dominated by charge interactions and salt bridges. In order to further investigate this we did an Ala scanning of the peptide using the Alphascreen assay (Fig. 1c). Our assay identifies three Ala mutations (V19A′, K20A′, L27A′) that affect the binding to PAC1R the most. The K20A′ mutation almost completely abolishes the ability of the peptide to bind the receptor ECD. The other important residues for binding are hydrophobic residues, Lue27′ and Val19′. This suggests that the major contribution to binding energy comes from a possible ionic interaction (K20′) that is supplemented by hydrophobic interactions mediated by V19′ and L27′. In the case of PTH1R, GIP1R, and CRFR1, peptide binding to ECD is also mediated by positively charged residues on the peptide, which is further stabilized by hydrophobic interactions from surrounding residues [27], [28]. Based on these observations, we attempted to derive the mechanism of PACAP binding. We initially started with a blind search for the peptide binding site in our structure using the essential region (residues 8–27) of the PACAP structure (residues 1–38, PDBId: 2D2P) in AutoDock 4.2 [38]. Guided by the putative peptide binding site, we chose a solution of the blind search for further examination. We then made the side chains of the peptide residues at the interface flexible and repeated the search with a finer grid. Our results are consistent with the hypothesis that PACAP8–27 docks with PAC1R at the putative binding site, as observed in other ECD:hormone complex structures. The peptide is oriented parallel to Helix 1. In this orientation, the N-terminal portion of the peptide is pointed towards the TM domain, consistent with its role in activation of the receptor. In contrast, the PACAP in the NMR structure is in a reverse orientation with its N-terminus pointing away from the TM domain of the receptor, further raising questions regarding the validity of the NMR structure.

**Figure 5:**
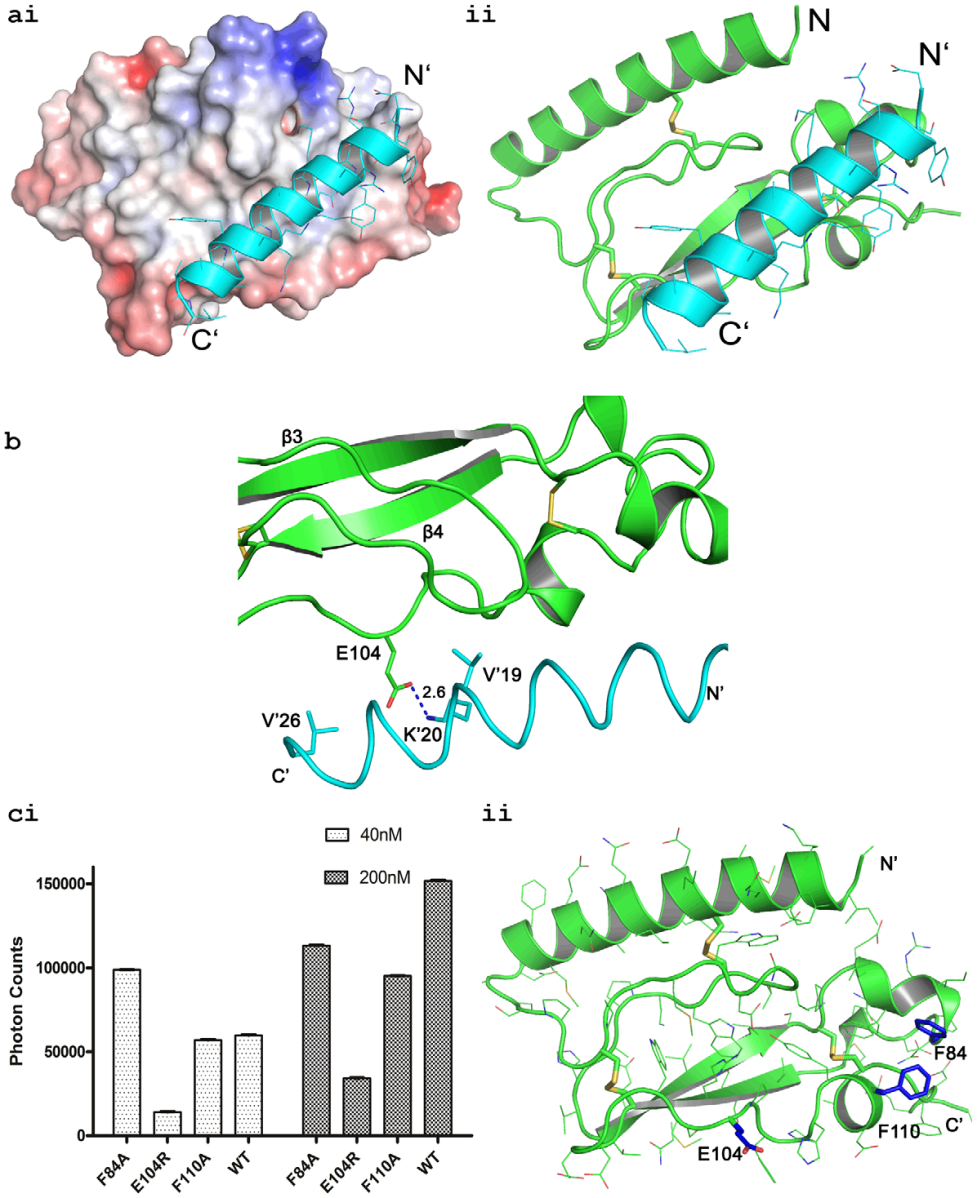
PAC1R and PACAP interaction model. (**a**) (**i**) The surface charge distribution of PAC1R is depicted with red and blue potentials, ranging from −10 KeT to +10 KeT. The potentials were calculated using APBS [47]. PACAP is shown in cyan. Our docking result correlates well with other published class B GPCR ECD:ligand complex structures. (**ii**) The polarity of the N-terminal α-helix of PAC1R and the PACAP is in the same direction. Following other structures, PACAP residues 1–8 are not expected to make any contact with the ECD of PAC1R and hence not included in our docking study. (**b**) A close-up view of the interaction. The residues that are likely to make important contacts are shown as sticks and are labeled. K20′ and E104 form a salt bridge with a distance of 2.6 Å. (**c**) (**i**) Alphascreen of mutations in PAC1R affecting the binding to PACAP. All the mutations were made on the outer surface of the receptor so that the structural core of PAC1R is unaffected. The mutants and the wildtype receptor ECDs were assayed for interaction with biotin-PACAP(6–38) at increasing equimolar concentration to enable the reading of the assay to reach saturation. (ii) The location of the mutated residues on the surface of PAC1R ECD. ECD is coloured in green while the side chains of the mutated residues are shown as blue sticks.

The docking model based on our X-ray structures correlates well with the binding data of alanine scanning peptides (Fig. 1c). The PACAP residues that are identified to be crucial for binding, according to the competition assay, are oriented towards the ECD, Fig. 5a, b. Val19′ and Leu27′ make hydrophobic interactions with the ECD. More importantly, Lys20′ makes a salt bridge with Glu104 of the ECD, which is the same conserved residue in CRFR1 that requires the positively charge residues (R35′) in CRF and urocortin 1 for the binding to the receptor [27], [28]. This relatively strong interaction appears to explain the ability of K20′A mutation to almost completely abrogate the peptide binding. This interaction implies that mutating E104 should also affect PACAP binding in a reciprocal manner. Fig. 5c shows that E104R mutation of the PAC1R ECD does reduce its binding to PACAP in an Alphascreen assay. In contrast, mutation in two surface hydrophobic residues (F84A and F110A) did not affect PACAP binding (Fig. 5c).The mutant ECDs were analysed for homogeneity in a manner analogous to the wild type ECD to ensure that the mutations do not affect the ECD's fold (Fig. S3).

### Mutational analysis in full length receptor system

**Figure 6:**
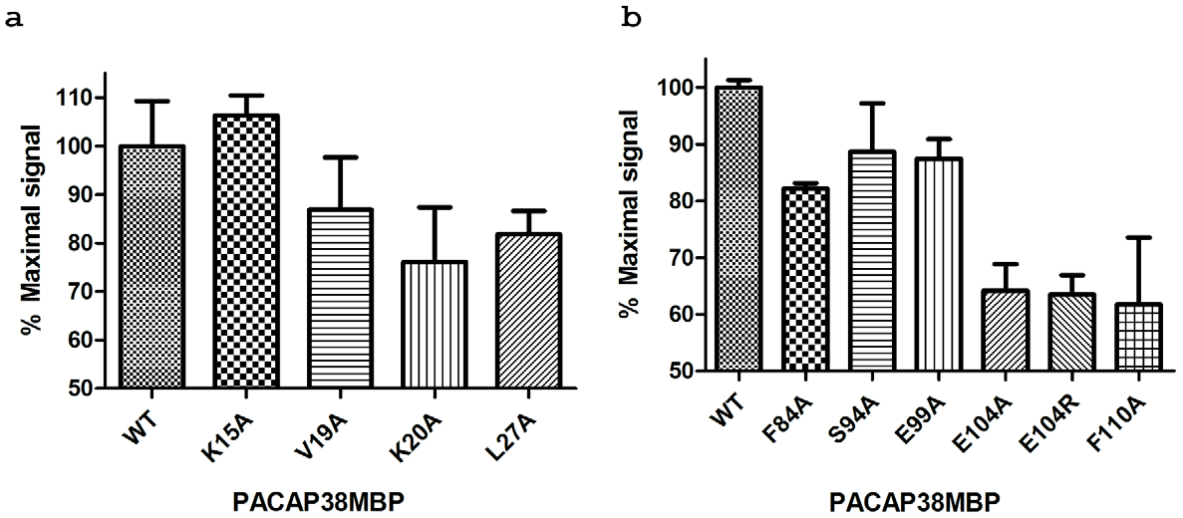
Stimulation of cAMP signaling by PACAP38-MBP. AD293 cells transiently transfected with PAC1R were stimulated with PACAP38-MBP for 4 hrs at 37°C after which the cells were lysed and assayed for cAMP content. All data points are average of duplicate samples and normalized to the stimulation level of wild-type receptor and peptide. (a) 0.03 nM PACAP38-MBP mutants were used to stimulate AD293 cells transiently expressing full length PAC1R-wt. (b) 0.03 nM PACAP38-MBP wt was used to stimulate cells transiently expressing PAC1R mutants.

To test our docking model in the full length receptor, we used a cAMP activated luciferase reporter in cell based assays. As shown in Figure 6 (a) and (b), mutations in the peptide/receptor interface affect PAC1R activation (L27′A, V19′A, and K20′A in the peptide; E104A, E104R, and F110A). In contrast, mutations outside of the peptide/receptor interface have little effects on the receptor activation (K15′A in the peptide; S94A and E99A in the receptor). Together with the ECD binding data, these results support the docking model of PACAP binding to the PAC1R ECD, and the exact intermolecular interactions between PACAP and PAC1R will require a high resolution structure of a PACAP∶PAC1R complex.

## Discussion

In this paper, we determined the crystal structure of the PAC1R ECD at 1.9 Å resolution, which reveals the same overall fold of other class B GPCR ECDs. Even though the NMR structure of PAC1R has been highlighted to have certain unique features among this class of receptors, there have been some differing views [44]. In our X-ray crystallographic structure, PAC1R follows the same conserved α+β fold. This fold is similar to the glucagon/VIP family due to the presence of a C-terminal helix. The previously reported NMR structure of PAC1R differs in the Cα tracing between β3 and β4 and displays a different topology of disulfide bond arrangement, as discussed in a review earlier [44]. The NMR structure of PAC1R is unlikely to be an alternate conformation in solution. Such an arrangement would be impossible without the disruption of the Cys77-Cys113 disulphide bond. The Cα atoms in the β3 and β4 loop have been recognised to be very important for ligand binding in other class B ECDs. In the complex structure of GIP1R, GLP1R (with both exendin-4 and glucagon) and PTH1R this loop forms a hydrophobic cluster. This hydrophobic cluster is the seat of hydrophobic interactions with the peptide in the complex structures of the above indicated ECDs. This cluster also contains the sequentially invariant Pro78, whose mutation in PTH1R leads to an embryonic lethal disorder. Therefore, the correct orientation of this loop is essential to structurally interpret PACAP binding. A different Cα trace in this region would change the hydrophobic pocket and would affect the binding of the peptide. In the NMR structure of PAC1R, PACAP makes no hydrophobic contacts in this region, making it unique in an otherwise unified class with respect to the hormone binding mechanism. Moreover, the polarity of PACAP in the NMR structure is opposite to all other known complex structures in this family. While the other structures report that the N-terminus of the peptide is roughly oriented towards the N-terminus of the ECD, in the NMR-PAC1R structure, the PACAP is oriented in an opposite way.

A two domain model of peptide binding to receptor has been suggested for class B GPCRs [21]. In this model, the C-terminal of the peptide first interacts with the ECD and this ‘affinity trap’ then helps the binding of the N-terminal part of the peptide with the juxtamembrane region of the receptor and activates the receptor. Based on our crystal structure, mutational analysis and the docking results, we propose a model for the PACAP binding to PAC1R. In this PACAP binding model, the PAC1R ECD adopts the same conserved fold as other class B GPCRs, and the PACAP peptide adopts a single helix that docks into the similar peptide binding site as observed in GLP1 and PTH peptides. This model highlights several critical features that are supported by structures and biology of Class B GPCRs. First, PAC1R belongs to the same subfamily of receptors as glucagon and GLP1. Thus, it is expected that their ECDs adopt the same topology in their structures as confirmed by their crystal structures [45], [46]. Second, PACAP also shares high degree sequence homology to glucagon and GLP1. Given this conservation and the conserved fold in their receptor ECD structures, it is reasonable to predict that PACAP adopts the same binding mode as GLP1 [46], therefore allowing its N-terminal residues to face the receptor TM domain to activate the receptor.

Finally, our proposed model is consistent with the mutagenesis data, including the PACAP alanine scanning peptides and mutation in the PAC1R ECD, particularly the K20A′ and E104R, respectively. These mutations stress a charge complementary mechanism for the binding of PACAP to PAC1R. The same charge complementary mechanism has also been revealed for a number of Class B GPCRs, including PTH1R, GLP1R, and CRFR1. Together, the results from our structural, biochemical, and modelling studies highlight a consensus structural fold for hormone recognition by Class B GPCRs, and should have important implications in hormone binding by several other members of Class B GPCRs, whose structures remain to be determined.

## Supporting Information

Figure S1 Sequence alignment of class B GPCRs. Invariant residues are highlighted and partially conserved residues are boxed. The secondary structural elements are shown for PAC1R. α-helices are drawn as helices and β-strands are drawn as arrows. PAC1R shares a significant level of sequence (33.1% identity) and structural similarity with VIP2R. Numbers are of PAC1R.
(TIF)

Figure S2 Cartoon of the overall PAC1R topology. The disulphide bonds are indicated as dashed lines.
(TIF)

Figure S3 Native PAGE of the refolded E105R mutant PAC1R ECD. The band of DsbC was removed by Ni chelate affinity chromatography and the minor upper bands were removed by size exclusion chromatography as described for the WT protein.
(TIF)
